# Linking customer big data analytics to firm performance: evidence from B2B organizations in an emerging market context

**DOI:** 10.3389/frai.2026.1757750

**Published:** 2026-05-05

**Authors:** Yasir Ali Soomro, Yasser Baeshen

**Affiliations:** 1 Carlton Trail College, Humboldt, SK, Canada; 2 Department of Marketing, Faculty of Economics and Administration, King Abdulaziz University, Jeddah, Saudi Arabia

**Keywords:** analytics culture, B2B marketing, customer big data analytics, customer satisfaction, firm performance

## Abstract

**Introduction:**

This study examines the impact of Customer Big Data Analytics (CBDA) on customer satisfaction and firm performance in business-to-business (B2B) firms operating in emerging markets, specifically Pakistan. Despite the growing adoption of big data technologies, empirical evidence on their strategic value in B2B contexts remains limited. This study also investigates the moderating role of analytics culture in strengthening these relationships.

**Methods:**

A quantitative research design was employed using survey data collected from 120 senior managers across multiple industries in Pakistan. The proposed conceptual model was tested using Partial Least Squares Structural Equation Modeling (PLS-SEM) to analyze the relationships among CBDA, customer satisfaction, firm performance, and analytics culture.

**Results:**

The findings indicate that CBDA has a significant positive effect on both customer satisfaction and firm performance. Customer satisfaction partially mediates the relationship between CBDA and firm performance. Additionally, analytics culture positively moderates the CBDA–firm performance relationship, suggesting that firms with a strong data-driven culture derive greater benefits from CBDA capabilities.

**Discussion:**

The study contributes to the B2B analytics literature by providing empirical evidence of the strategic importance of CBDA and analytics culture in emerging markets. The results highlight the need for firms to not only invest in data analytics capabilities but also foster a supportive analytics culture to maximize performance outcomes. Practical implications are discussed for managers aiming to enhance customer satisfaction and firm performance through effective use of big data analytics.

## 1 Introduction

Recent empirical studies reinforce the view that analytics-driven resources transform into superior business value when embedded in complementary capabilities and culture within firms. In B2B contexts, customer analytics capability has been shown to emerge from a complementarity between outside-in and inside-out resources, and this capability later improves firm performance; an effect that varies by firm age (Keramati et al., 2024). Moreover, organizational environment/analytics culture has been identified as a critical boundary condition that shapes how Big Data Analytics (BDA) capabilities convert to innovation quality and speed, highlighting that culture is not merely an enabler but a significant amplifier of analytics value (Foroughi and Iranmanesh, 2024). A study also explains the mechanism linking BDA to performance: a meta-analysis study finds that organizational agility acts as a proven mediator between BDA and firm performance, with national culture shaping the BDA and agility (Liu et al., 2025). Together, these findings expand the Resource-Based View by emphasizing capability complementarity, cultural context, and agility-based transmission mechanisms, hence they motivate research focus on customer-centric BDA, customer satisfaction, and analytics culture in B2B firms. Business operations such as supply chain and customer relationship management are strengthened by big data analytics (Gunasekaran et al., 2017; Nam et al., 2019; Zerbino et al., 2018). It is perceived that sales and customer services will be personalized as well as customized through the increasing use of big data analytics in context of customer relationship management (Anshari et al., 2019). Big data analytics will also help to develop strong personal relationship with consumers as well as customers (de Lima Francisco et al., 2016). Big data will be useful in understanding customer expectations and their ever-changing future demands (Perera et al., 2018) through the usufruct of big data tools and technologies (Emtiyas and Keyvanpour, 2011).

Business knowledge is created through the exploitation of big data (Davenport, 2014), which refers to the understanding and information availability related to business processes and environment (Wang et al., 2016) that helps corporations in decision-making (Bharadwaj et al., 2013; Chen et al., 2014). To optimize the understanding of customer preferences and business processes, big data analytics plays a pivotal role in reaching fact-based decision-making (Wamba et al., 2017). Big data analytics helps to undermine the obscure behavioral patterns of customers (Erevelles et al., 2016). The key features of BDA can be identified as the creation of real-time knowledge of markets, the use of real-time data, and the provision of instant information (Xu et al., 2016). If these are utilized properly, they can lead to an increase in sales. Profound customer information can be obtained through the use of BDA, but to capitalize on this information to acquire market advantage is a challenge faced by marketers (Erevelles et al., 2016). Prior study reveals that investment in big data does produce results for the firm, but analytics culture is an important catalyst to achieve competitive advantage (Hallikainen et al., 2020).

In the current B2B marketing literature, Wiersema (2013) identifies big data analytics as one of the emerging topics and the main source of competitive advantage. Recent technological developments have made data collection increasingly practical and cost-efficient (Frizzo-Barker et al., 2016). Organizations now have access to extensive information generated through both internal operations and external digital sources (Lilien, 2016). Although big data analytics holds significant promise for B2B firms, many practitioners still lack the appropriate tools, skills, and strategic guidance needed to fully harness its potential (Lilien, 2016). At the same time, many firms remain uncertain about how big data can improve their business processes (Swanson and Ramiller, 2004). or what value such initiatives may generate (Lycett, 2013), and the academic community has yet to provide clear direction on these questions (Frizzo-Barker et al., 2016). As highlighted in recent research (Sivarajah et al., 2017; Wang and Hajli, 2017), this gap exists partly because scholarly work on big data is still emerging, with much of the existing discussion focused on technical experiments and simulation-based approaches rather than managerial and strategic implications. As a result, there is a lack of empirical evidence demonstrating how big data analytics influences customer satisfaction and firm performance in real organizational settings.

To address this gap, the present study investigates the central question: How does customer big data analytics enable B2B firms to enhance customer satisfaction and ultimately improve firm performance? This study examines the effects of customer big data analytics on customer satisfaction and firm performance. It also proposes that an analytics-oriented culture is a significant moderating factor in the relationship between customer big data analytics and firm performance. The underlying assumption is that firms with a strong analytics culture, where employees value data-driven decision-making and perceive clear benefits from analytics, are more likely to generate greater customer satisfaction and stronger performance outcomes from big data initiatives than firms without such a culture.

This study makes several contributions to the academic body. First, empirical research specifically examining how customer big data analytics (CBDA) impacts the B2B Firm Performance, particularly within the Pakistani context, remains limited. Second, while prior work suggests that an analytics culture is important in the context of competitive advantage and enhancing the benefits of big data analytics (Kiron et al., 2014), there is a need for empirical validation to understand the role that analytics culture plays in customer satisfaction and firm performance. Third, this study provides important empirical evidence about the role of customer big data analytics on customer satisfaction moderated by analytics culture within B2B settings, as B2B organizations continue to lag behind B2C firms in adopting and capitalizing on big data analytics (Lilien, 2016). This reflects the growing interest in academic literature in understanding how big data analytics shapes strategic decision-making and business outcomes.

## 2 Literature review

The question that needs to be investigated is whether big data analytics (BDA) can create value for enterprises to acquire competitive advantage and if so, when, why, and how this value is generated. This issue can be addressed through extended research furthering from the post adoption stage (LaValle et al., 2011; Agarwal and Dhar, 2014; Abbasi et al., 2016; Xu et al., 2016; Côrte-Real et al., 2017). The literature suggests that BDA outcomes are critical components used to transform data into meaningful business insights, which ultimately translate into improved business performance (Raguseo and Vitari, 2018). BDA due to its strategic competence and extraordinary operations, is recognized as a trend setter which improves business effectiveness and efficiency (Wamba et al., 2017). A recent study conducted by Ji-Fan Ren et al. (2016) has tried to dwell upon the association of BDA solutions and company performance.

### 2.1 Theoretical foundations and research hypotheses

It is observed that BDA and firm performance are now being focused more on evaluating business value (McAfee et al., 2012; Kiron et al., 2014; Akter et al., 2016). Based on prior studies, the outcomes of BDA can be categorized into four types of value creation: transformational, transactional, strategic, and informational value (Gregor et al., 2006).

Transactional value refers to operational advantages derived from BDA, such as reductions in operating costs, improvements in employee productivity, and enhanced efficiency in supply chain management. These operational improvements enable organizations to estimate and enhance the capacity generated through BDA utilization.

Strategic value arises when BDA supports improvements in product innovation and customer service quality, thereby enhancing the firm’s offerings to its customers. Informational value relates to an organization’s ability to improve information flow, enabling faster and easier access to data while converting raw data into usable and meaningful formats.

When organizations strive to achieve competitive advantage, two key factors play a pivotal role: capitalizing on emerging market opportunities and restructuring existing business models. The value created by BDA in supporting these activities is often referred to as transformational value. In this context, the collection, availability, and effective utilization of data become essential in today’s business environment. Consequently, BDA enhances information flows and enables faster and more user-friendly access to timely data.

Prior research also categorizes various big data applications based on two dimensions: time horizon and scale (Matthias et al., 2017). The scale dimension refers to the breadth of application. Macro-level applications focus on large organizational domains, such as the management of an entire supply chain (Wang et al., 2016). In contrast, micro-level applications focus on specific business operations or individual processes, such as customer recommendation systems.

The time horizon dimension refers to how BDA is applied across temporal perspectives. When data is analyzed using descriptive, predictive, or prescriptive analytics, organizations can better understand past, present, and future business conditions (Wang et al., 2016). For example, historical data may be used for management control and auditing, providing descriptive insights. Current data can support prescriptive decision-making through real-time responses. Future-oriented analysis enables organizations to identify strategic opportunities and potential risks (Gunasekaran et al., 2017). Based on these dimensions, six types of analytics applications emerge, combining macro and micro scales with past, present, and future time horizons (Matthias et al., 2017).

This classification helps explain why BDA can generate value across multiple stages of the enterprise value chain. BDA can accelerate strategic improvements and operational efficiency (Wang et al., 2016), support automation and operational optimization (Davenport, 2014; Wamba et al., 2015), enhance organizational agility and flexibility (Wang et al., 2016), improve information management (Côrte-Real et al., 2017), strengthen managerial decision-making processes (Davenport, 2014; Wang et al., 2016), and improve the architecture of information technology systems (Wang et al., 2016). Furthermore, BDA enables greater transparency and data reliability (Wamba et al., 2015) and allows firms to better segment customers based on demographic and behavioral characteristics (Wamba et al., 2015).

In practice, firms can achieve competitive advantages by leveraging BDA capabilities across various stages of data utilization (Côrte-Real et al., 2017). However, organizations must implement appropriate managerial processes to ensure that investments in big data lead to sustainable competitive advantages. These processes allow firms to identify the resources needed to develop specialized capabilities, overcome implementation barriers, and ultimately achieve strategic success (Wang et al., 2016; Braganza et al., 2017).

Compared with traditional database marketing approaches, big data analytics enables firms to transform customer data into actionable knowledge that can be scaled efficiently and securely to create business value. As a result, big data–enhanced database marketing offers stronger prospects for business-to-business (B2B) customer relationship management (Orenga-Roglá and Chalmeta, 2016). Big data analytics provides valuable intelligence for marketers and enables firms to develop more customer-focused strategies (Orenga-Roglá and Chalmeta, 2016), allowing them to respond quickly to changing customer preferences and needs (Xu et al., 2016). For example, BDA enables more personalized pricing strategies, product recommendations, and tailored offerings (Martin and Murphy, 2017). Consequently, firms can build stronger customer relationships and operate in a more customer-oriented manner (Erevelles et al., 2016).

Prior research indicates that BDA has strong potential to improve organizational performance (Wamba et al., 2015, 2017; Akter et al., 2016), and its effective utilization can lead to significant competitive advantages (Matthias et al., 2017).

Many organizations continue to seek a deeper understanding of how big data analytics can be effectively integrated into business operations (Lycett, 2013). Drawing on the resource-based view of the firm (Wernerfelt, 1984; Kozlenkova et al., 2014), previous research suggests a direct relationship between information technology investments and firm performance (Huang et al., 2006; Melville et al., 2004), as well as between investments in big data analytics and firm performance (Akter et al., 2016; Nam et al., 2019; Wamba et al., 2017; Wang and Hajli, 2017). Furthermore, studies show that aligning business strategy with analytics capabilities strengthens a firm’s ability to utilize big data analytics effectively and improve organizational performance (Akter et al., 2016).

Customer big data analytics enables firms to utilize real-time customer data and respond quickly to changing customer demands (Xu et al., 2016). In the context of customer relationship management, such capabilities can significantly improve market performance and financial outcomes.

Market performance refers to a firm’s ability to enter new markets faster than competitors, develop innovative offerings more frequently, achieve higher success rates with new products and services, and capture greater market share. These factors often contribute to stronger financial outcomes. For example, entering new markets may generate higher returns and improved profitability, thereby influencing a firm’s financial performance (Homburg et al., 2007). Similarly, the introduction of innovative products and services can increase sales and create positive word-of-mouth among customers, thereby improving financial performance (Szymanski and Henard, 2001).

Given the potential influence of market performance on financial outcomes, it becomes important to examine whether market performance mediates the relationship between the business value of BDA solutions and firm profitability. Previous research has explored similar relationships in various contexts (Chumpitaz and Paparoidamis, 2004). Scholars also emphasize that big data should be integrated into new product development and strategic marketing processes in order to fully realize its potential in marketing contexts (Tan et al., 2015; Wamba et al., 2015; Xu et al., 2016). This integration may help firms increase their market influence and capture greater shares of existing markets (Wamba et al., 2017). Applications of BDA may permit firms identify market opportunities, promote organizational agility, modify the firm’s products and services and captivate possible market chances (Côrte-Real et al., 2017). Lastly, another innovative type of commercial approach that leverages on informatization practices is possibly a disruptive establishment of new streams of revenue generations by trading of data that is contrary to the customary offerings of goods and services (Opresnik and Taisch, 2015). In line with Wamba et al. (2017), this study indicates that the utilization of customer big data analytics (CBDA) improves firm performance. In this study operationalized through two dimensions: market performance and financial performance. Accordingly, this leads to the first hypothesis of the study:

*H1*: Customer big data analytics positively affects firm performance.

### 2.2 The mediating effect of customer satisfaction

Customer satisfaction refers to how well a product or service meets or exceeds buyers’ expectations. If perceived performance surpasses expectations, buyers are content; if it falls short, they are dissatisfied. Research suggests that customer satisfaction influences customer loyalty and, in turn, company profits (Anderson and Sullivan, 1993). Several studies (Farooq et al., 2018; Soomro et al., 2022) highlight that achieving high customer satisfaction is essential for long-term business effectiveness, sustainability, and loyalty (Soomro et al., 2022). Further, satisfied customers drive better financial performance in both manufacturing and services, with established links between satisfaction, loyalty, and financial outcomes (e.g., Chi and Gursoy, 2009). Increasing customer satisfaction is associated with a better understanding of customer needs through CBDA solutions, leading to greater loyalty and improved future cash flows. Companies can use big data to further enhance satisfaction (Wamba et al., 2017). Recent research links big data analytics (BDA) to increased company efficiency and reduced customer acquisition costs (Liu, 2014), stronger customer relationships (Cheng et al., 2016), better fulfilment of specific needs (Wamba et al., 2015), and higher overall satisfaction (Tan et al., 2015). Ultimately, customer satisfaction may act as a key intermediary between the value derived from CBDA solutions and an enterprise’s financial performance. This forms the basis for our second and third hypotheses, as shown below:

*H2*: Customer big data analytics positively affects customer satisfaction.

*H3*: Customer satisfaction positively affects firm performance.

*H4*: Customer satisfaction mediates the relationship between customer big data analytics and firm performance.

### 2.3 Moderating role of analytics culture

An analytics culture depicts the shape of mutual values and beliefs inside a firm (Germann et al., 2013), and it brings together technology and business across a common field through a specific set of decision-making norms, behaviours, outcomes, and values (Kiron et al., 2014). Germann et al. (2013) recommend that the analytics culture of a company plays a pivotal role in incorporating perceptions acquired from marketing analytics to influence a business’s decision-making. The effectiveness of such analytics initiatives often depends on the organizational context in which they are embedded. Prior research highlights that analytics initiatives do not operate in isolation; rather, their success depends on the organizational culture that supports data-driven decision-making (Germann et al., 2013). In view of big data analytics, Kiron et al. (2014) imply that an analytics culture serves as an enabler pushing a firm from competitive equality to a competitive lead when it comes to creating additional proceeds from customer big data analytics. Moreover, the analytics culture of an organization here implies the degree to which a corporation is encouraging marketing analytics to be used within the firm (Germann et al., 2013; Hallikainen et al., 2020) and fosters data-backed decision making. Analytics culture is expected to strengthen the relationship between customer big data analytics and customer satisfaction. Similarly, analytics culture is anticipated to amplify the positive effect of customer big data analytics on firm performance (H5b). Firms with strong analytics cultures are better positioned to operationalize insights, optimize processes, enhance customer targeting, and make informed strategic decisions. Prior studies suggest that analytics-oriented cultures help firms move from competitive parity to sustained competitive advantage by enabling more effective use of big data (Kiron et al., 2014). Based on the reasons provided, following hypotheses were created:

*H5a*: Analytics culture positively moderates the relationship between customer big data analytics and customer satisfaction.

*H5b*: Analytics culture positively moderates the relationship between customer big data analytics and firm performance.

### 2.4 Proposed conceptual research model

See Figure 1.

**Figure 1:**
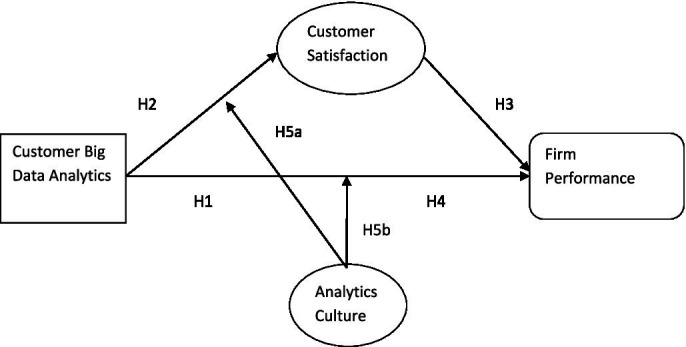
Hypothesized research model.

## 3 Data and method

To address the research objectives and test the hypotheses, we developed a structured questionnaire for CEOs and senior-level managers. These respondents were chosen for their direct knowledge of strategic decisions, customer management, and big data analytics in their firms. Authors analyzed the data using partial least squares structural equation modeling (PLS-SEM), including latent moderated structural models, to evaluate the measurement and structural components of the research framework.

### 3.1 Measurement scales, data collection, and sample

All measurement items used in the questionnaire were adapted from well-established and validated scales in prior research. Customer big data analytics was measured using a seven-item scale derived from Jayachandran et al. (2005) and Hallikainen et al. (2020) Although originally developed in a CRM context, the scale captures firms’ capability to collect, integrate, and utilize customer information for strategic decision-making. The items were contextualized to reflect contemporary big data analytics environments and align with recent research conceptualizing customer analytics capabilities in B2B settings (Hallikainen et al., 2020). original items were slightly refined to specifically capture the use of customer big data analytics rather than general customer information practices. Customer satisfaction was measured using four items adopted from Vorhies and Morgan (2005) and Mithas et al. (2011). Firm performance was conceptualized as a combination of market performance and financial performance, with items adapted from Maslowsky et al. (2015) and Ji-Fan Ren et al. (2016). Respondents evaluated their performance relative to competitors, reflecting both relational (non-financial) and financial outcomes. Analytics culture, the moderating construct, was measured using the widely accepted three-item scale developed by Germann et al. (2013), which captures the extent to which organizations support and value the use of analytical insights in decision-making.

All multi-item constructs were measured on a five-point Likert scale. For customer big data analytics and analytics culture, response options ranged from 1 = strongly disagree to 5 = strongly agree. For customer satisfaction and firm performance measures, respondents rated their firm’s performance using a scale of 1 = Much worse to 5 = Much better, relative to their competitors.

The implementation of customer big data analytics often requires significant technological resources and organizational capabilities (Erevelles et al., 2016; Xu et al., 2016), authors employed a purposive key-informant sampling approach targeting CEOs, owners, and senior managers in firms with at least 10 employees, as such respondents were expected to possess sufficient knowledge of customer analytics practices and firm performance. A private business database served as the sampling frame, and survey invitations were sent by email over a three-week period. A total of 200 invitations were distributed. Authors received 150 questionnaires back that were complete and usable. Authors requested respondents to identify that their firm focuses specifically on B2B business model, 30 responses came from firms that also deal with B2C and they were excluded from sample, resulting in a final analytical sample of 120 B2B firms. Screening criteria required that respondents occupy decision-making roles, that firms have at least 10 employees, and that firms operate primarily in a B2B context. To assess non-response bias, early and late respondents were compared on key study variables, and no significant differences were observed, suggesting that non-response bias was not a major concern. Pakistan was selected as the empirical setting because it offers an important emerging-market context in which B2B firms are increasingly adopting analytics-driven practices, while evidence on the performance implications of such capabilities remains limited.

Although the final sample size is modest with 120 samples, it is acceptable for the present study. In PLS-SEM, sample size adequacy is commonly assessed in relation to the maximum number of structural paths directed at any endogenous construct (Hair et al., 2021). In the current model, the most complex endogenous construct is firm performance, which has three incoming predictors. Therefore, the sample of 120 exceeds the minimum requirement suggested by commonly used PLS-SEM guidelines. Prior multivariate research suggests that sample size adequacy in multivariate models should be considered in relation to model complexity and statistical power; as a rule of thumb, 10 observations per predictor may represent a minimum, whereas larger samples such as 30 observations per predictor are preferable when smaller effects are expected (VanVoorhis and Morgan, 2007), a sample size of 120 also reflects more than 20 observations per construct, which further supports the adequacy of the sample for the present model. Taken together, these considerations indicate that the sample size is adequate for estimating the proposed model.

The data collected for the exogenous, mediator, moderator, and endogenous variable were collected through a survey instrument from respondents, hence common method bias (CMB) was a potential concern to be addressed. Several procedural and statistical remedies were considered. Procedurally, respondents were explicitly informed that their responses would remain confidential and be only used for academic purposes, this helped reduce evaluation apprehension and social desirability bias. Moreover, the questionnaire relied on established multi-item scales adapted from prior research and was structured to improve clarity and reduce ambiguity. Statistically, authors examined the outer, inner and full collinearity variance inflation factor (VIF) values and all were under the accepted range.

Table 1 presents the demographic profile of the respondents. The sample is predominantly male (80%), with the majority occupying senior or middle management roles. Firms in the dataset represent key industries such as manufacturing, IT and software, digital marketing, and financial services. Most participating firms fell within the medium-sized enterprise category, followed by large organizations.

**Table 1:** Respondent demographics.

Measure	Frequency	%
Gender		
Female	24	20
Male	96	80
Job level		
Senior management	51	42.5
Middle management	45	37.5
Entrepreneur	24	20
Industry		
Digital marketing agencies	15	12.5
Manufacturing	60	50
IT and software	30	25
Banks and financial institutions	15	12.5
Size of the company		
Medium	75	62.5
Large	45	37.5

*n* = 120.

## 4 Results

### 4.1 Partial least square structural equation modeling

To analyze the conceptual model, a two-stage approach of PLS-SEM was applied which included measurement and the structural model assessment. PLS-SEM was selected because the study aims at prediction and theory extension, and because the final sample size of 120 is adequate relative to model complexity.

#### 4.1.1 Measurement model assessment

The reliability and validity of the measurement model were examined prior to testing the structural relationships. As shown in Table 2, all constructs demonstrated strong internal consistency reliability. Cronbach’s alpha values for the constructs exceeded the recommended threshold of 0.60, with many constructs surpassing 0.80, indicating robust reliability consistent with the guidelines of Hair et al. (2014). Composite reliability (CR) values were also above the acceptable cut-off of 0.70 (Fornell and Larcker, 1981), suggesting that the items adequately captured the underlying latent constructs.

**Table 2:** Measurement model assessment.

Variables and source	Items	FL	CA	CR	AVE	Outer VIF
Customer big data analytics (Jayachandran et al., 2005; Hallikainen et al., 2020)	Our company uses big data…		0.848	**0.864**	**0.765**	
	CBDA1: to develop customer profiles.	0.893				2.321
	CBDA2: to segment markets.	0.859				2.467
	CBDA3: to assess customer retention behavior.	0.859				2.432
	CBDA4: to identify appropriate channels to reach customers.	0.831				2.868
	CBDA5: to customize offers.	0.745				2.164
	CBDA6: to identify our best customers.	0.848				2.529
	CBDA7: to assess the lifetime value of our customers.	0.776				1.933
Customer satisfaction (Vorhies and Morgan, 2005; Mithas et al., 2011)	Our company has achieved…		**0.787**	**0.741**	**0.696**	
	CS1: higher customer satisfaction	**0.841**				2.136
	CS2: delivered more value to our clients	**0.786**				2.104
	CS3: improved delivery of what our clients require	**0.834**				2.249
	CS4: retained valued customers to a greater extent	**0.778**				1.847
Firm performance *Market performance* (Ji-Fan Ren et al., 2016) *Financial performance* Mithas et al. (2011)	Our company has been able to …		**0.812**	**0.843**	**0.712**	
	MP1: enter new markets more quickly than our competitors	**0.748**				1.864
	MP2: introduce new products and services to our clients faster than our competitors	**0.708**				1.932
	MP3: achieve higher success rate of new products or services than our competitors	**0.720**				2.041
	MP4: achieve greater market share than our competitors	**0.768**				2.276
	FP1: achieved growth in sales as compared to last year’s figures relative to our competitors.	**0.767**				2.491
Analytics culture (Germann et al., 2013)	AC1: If we reduce our marketing analytics activities, our company’s profits will suffer.	**0.785**	**0.688**	**0.741**	**0.812**	2.608
	AC2: We are confident that the use of marketing analytics improves our ability to satisfy our customers.	**0.767**				2.355

FL, Factor loadings; CA, Cronbach’s Alpha; CR, composite reliability; AVE, average variance extracted. All constructs demonstrate acceptable reliability and validity. All measurements meet recommended threshold values.

Convergent validity was assessed through factor loadings and average variance extracted (AVE). All factor loadings were greater than 0.70, demonstrating that the indicators strongly represented their respective constructs. In addition, the AVE values for all constructs were above 0.50, confirming that more than half of the variance in the indicators was explained by the constructs, thereby establishing adequate convergent validity. All VIF values of items are below 5.0, indicative of no multicollinearity among indicators. These results collectively indicate that the measurement model possesses sound psychometric properties.

Discriminant validity was evaluated using the Fornell and Larcker (1981) criterion. Table 3 shows that the square root of the AVE for each construct exceeded its correlations with other constructs, confirming that each construct is empirically distinct from the others. Thus, the requirements for discriminant validity were satisfactorily met, strengthening confidence in the measurement model.

**Table 3:** Discriminant validity assessment.

Construct	Customer big data analytics	Customer satisfaction	Firm performance	Analytics culture
Customer big data analytics	**0.841**			
Customer satisfaction	0.098	**0.723**		
Firm performance	0.455	0.652	**0.914**	
Analytics culture	0.375	0.271	0.426	**0.886**

Diagonal elements shown in bold represent the square root of the Average Variance Extracted (AVE), while off-diagonal elements represent inter-construct correlations.

In the structural model, the inner VIF values ranged from 2.214 to 2.774, which are below 5.0, suggesting no collinearity issue in the structural model. Moreover, full collinearity VIF values were examined to assess potential common method bias. All the values were below the recommended threshold of 3.3, hence common method bias is unlikely to be a serious concern and unlikely to affect the validity of the results (see Tables 4, 5).

**Table 4:** Inner VIF values (structural model).

Predictor	Customer satisfaction	Firm performance
Customer big data analytics	2.214	2.486
Customer satisfaction		2.631
Analytics culture × CBDA	2.357	2.774

**Table 5:** Full collinearity VIF values (common method bias test).

Construct	Full collinearity VIF
Customer big data analytics	2.214
Customer satisfaction	2.487
Analytics culture	1.936
Firm performance	2.774

#### 4.1.2 Structural model assessments

After the reliability and validity of the measurement model, the structural model was assessed to evaluate the hypothesized relationships. A baseline model (Model 1), which included only the direct effects, was first estimated. The results (see Table 6) show that customer big data analytics had a positive and significant effect on firm performance (*β* = 0.779, *p* < 0.001) and on customer satisfaction (*β* = 0.777, *p* < 0.001), providing strong support for H1 and H2. Furthermore, customer satisfaction had a significant positive effect on firm performance (*β* = 0.562, *p* < 0.001), supporting H3. These findings indicate that firms leveraging customer big data analytics tend to achieve higher levels of customer satisfaction, which subsequently enhances firm performance.

**Table 6:** Structural model results.

Relationship paths	Standard beta	*t*-value	p*-*values	Conclusion
H1. customer big data analytics → firm performance	0.779	7.249	0.000	Supported
H2. customer big data analytics → customer satisfaction	0.777	5.157	0.000	Supported
H3. customer satisfaction → firm performance	0.562	7.676	0.000	Supported

#### 4.1.3 Mediation analysis

To test the mediating role of customer satisfaction, an indirect effects analysis was conducted. The mediation results (see Table 7) confirm that customer satisfaction partially mediates the relationship between customer big data analytics and firm performance (*β* = 0.432, *t* = 11.637, *p* < 0.001). This means that customer big data analytics improves firm performance both directly and through its positive influence on customer satisfaction. The presence of partial mediation suggests that while analytics capabilities directly enhance performance outcomes, an important pathway also operates through increased customer satisfaction.

**Table 7:** Mediation analysis (indirect effect).

Relationship path	Standard beta	*t*-value	p*-*values	Conclusion
H4. customer big data analytics → customer satisfaction → firm performance	0.432	11.637	0.000	Partial mediation

#### 4.1.4 Moderation analysis

Hypothesis 5a proposed that analytics culture would strengthen the relationship between customer big data analytics and customer satisfaction. This hypothesis was tested using latent moderated structural equations. The interaction term between customer big data analytics and analytics culture was positive and significant (*β* = 0.095, *t* = 2.650, *p* < 0.001), supporting H5a. The positive effect of big data analytics on customer satisfaction is amplified when firms possess a strong analytics culture, indicating that the internal environment of the firm plays a crucial role in maximizing the value derived from data analytics initiatives.

Similarly, Hypothesis H5b predicted a moderating effect of analytics culture on the relationship between customer big data analytics and firm performance. The results in Table 8 support this hypothesis as well (*β* = 0.098, *t* = 2.793, *p* = 0.005). Firms with a well-developed analytics culture experience a stronger positive impact of big data analytics on performance compared to firms with weaker analytics cultures. Satorra–Bentler scaled chi-square difference tests further verified that including the interaction term significantly improved model fit (Δ*χ*^2^(1) = 24.992, *p* < 0.001), confirming the relevance of the moderation.

**Table 8:** Moderation analysis (interaction effect).

Relationship paths	Standard beta	*t*-value	p*-*values	Conclusion
H5a. customer big data analytics *X* analytics culture → customer satisfaction	0.095	2.650	0.008	Supported
H5b. customer big data analytics *X* analytics culture → firm performance	0.098	2.793	0.005	Supported

## 5 Discussion

The findings of this research confirm that Customer Big Data Analytics (CBDA) serves as a critical strategic resource for improving firm performance in B2B organizations. CBDA enables firms to acquire valuable, rare, and inimitable insights into customer behaviors and market conditions that enhance decision-making and operational agility.

The results indicate a strong positive relationship between CBDA and firm performance, affirming earlier studies (Akter et al., 2016; Wamba et al., 2017), which argue that analytics-driven organizations enjoy improved profitability and growth. The ability to translate vast customer data into actionable intelligence contributes to market expansion, product innovation, and enhanced competitiveness. The results are consistent with and extend emerging evidence that the route from CBDA to performance runs through adaptive capabilities. Meta-analytic findings show that CBDA consistently boosts organizational agility, which in turn enhances firm performance, and that contextual factors (e.g., national culture) condition these effects (Liu et al., 2025). Recent B2B research further indicates that customer-facing analytics capability is strongest when firms align outside-in market orientation with inside-out data-driven culture (Keramati et al., 2024), while hybrid empirical designs (PLS + fsQCA) reveal that analytics culture and BDA capability combine configurationally to drive innovation outcomes (Foroughi and Iranmanesh, 2024). Looking ahead, adjacent literatures tie BDA capabilities to sustainability-oriented outcomes (e.g., green innovation), suggesting promising cross-domain benefits and new mediators beyond customer satisfaction (El Manzani et al., 2025). These insights reinforce our recommendation to invest not only in tools but also in culture, agility, and customer analytics capability building.

A key theoretical contribution lies in demonstrating that customer satisfaction partially mediates the relationship between CBDA and firm performance. This finding implies that while analytics enhances performance directly, its most substantial effects are realized when insights are used to improve customer value and service personalization. These results align with previous studies (Anderson and Sullivan, 1993; Chi and Gursoy, 2009), which found that satisfied customers are more loyal, generate repeat business, and amplify positive word-of-mouth; all critical drivers of firm success in B2B contexts. Moreover, the moderating role of analytics culture underscores that technology alone is insufficient; organizational values, norms, and leadership commitment to data-driven decision-making determine how effectively analytics can translate into tangible outcomes. Firms exhibiting a strong analytics culture experienced a significantly stronger relationship between CBDA and performance, confirming the propositions of Germann et al. (2013) and Kiron et al. (2014). This finding suggests that cultivating a culture of analytical thinking and continuous learning magnifies the strategic benefits of CBDA.

## 6 Conclusion

The main objective of this study was to examine how Big Data Analytics (BDA) influences customer satisfaction and firm performance within business-to-business (B2B) organizations, and how analytics culture strengthens this relationship. CBDA constitutes a valuable, rare, and hard-to-imitate organizational capability that enables firms to transform large volumes of customer and market data into actionable insights. Empirical analysis using Partial Least Squares Structural Equation Modeling (PLS-SEM) confirmed that CBDA exerts a significant positive effect on both customer satisfaction and overall firm performance. This finding provides strong empirical support for the argument that data-driven decision-making enhances organizational agility, operational efficiency, and strategic responsiveness.

A key contribution of this study is the identification of customer satisfaction as a mediating mechanism linking CBDA and firm performance. The results suggest that the value of analytics does not lie merely in data collection or processing but in the firm’s ability to translate analytical insights into superior customer experiences. When firms use CBDA to anticipate customer needs, personalize interactions, and improve service delivery, customer satisfaction rises, leading to stronger retention rates, repeat purchases, and enhanced profitability. Thus, the findings highlight that customer satisfaction represents the primary conduit through which big data creates tangible business value in B2B markets.

Equally important, the study provides robust evidence for the moderating role of analytics culture. Firms characterized by strong analytical cultures, where data-based reasoning is encouraged, performance metrics are transparent, and employees value evidence-driven decisions that derive substantially greater benefits from CBDA. This suggests that analytics culture acts as a catalyst, transforming technical analytics capabilities into strategic assets. Without such a culture, even advanced data systems may fail to influence decision-making or improve outcomes. The findings, therefore, underscore that technological investment must be complemented by managerial commitment to foster a pervasive data-driven mindset across all levels of the organization.

## 7 Implications and limitations

From a managerial standpoint, the study implies that leaders in B2B firms should view big data initiatives as strategic transformations rather than as isolated technological projects. To realize the full potential of CBDA, firms must invest not only in analytical tools but also in human capital development, cross-functional collaboration, and a culture that rewards experimentation and learning. In markets where competition is increasingly data-intensive, such holistic integration of analytics and culture can differentiate high-performing firms from those that merely adopt technology without strategic alignment.

From an academic perspective, this research extends existing literature by integrating both mediating (customer satisfaction) and moderating (analytics culture) mechanisms into a unified model, providing a richer understanding of how CBDA enhances performance in B2B settings. It contributes empirical evidence from an emerging-market context, addressing a gap in prior studies that have focused predominantly on developed economies. By validating the conceptual linkages among CBDA, satisfaction, and firm outcomes, the study advances theoretical discourse and offers a replicable framework for future investigations.

In conclusion, this study establishes that Customer Big Data Analytics (CBDA) is not merely an operational tool but a strategic capability that can generate sustained competitive advantage when supported by a strong analytics culture and customer-centric orientation. Firms that successfully integrate data insights into strategic and relational decisions are more likely to achieve superior performance outcomes and long-term business success. Ultimately, the intersection of analytics capability, organizational culture, and customer orientation represents the next frontier for competitive differentiation in the evolving landscape of B2B marketing and management.

Despite the valuable insights generated by this study, several limitations should be acknowledged. First, the research was conducted within a single national context of Pakistan, which may limit the generalizability of the findings to other developed countries with different cultural, economic, or technological conditions. Future replications across multiple countries would help determine whether the observed relationships between big data analytics, customer satisfaction, and firm performance hold consistently across diverse contexts. Second, the study employed a cross-sectional design, capturing data at one point in time, which restricts the ability to infer causal relationships, hence the findings should be interpreted as associational, and the long-term effects of customer big data analytics on customer satisfaction and firm performance cannot be fully assessed or observe. A longitudinal approach could offer deeper insight into how firms evolve as they mature in their analytics capabilities. Third, the reliance on self-reported data from senior managers introduces potential response bias, as participants may overestimate their firm’s analytics proficiency or performance outcomes, though authors applied procedural and statistical methods to address common method bias. It is suggested to conduct Harman’s single-factor test. Fourth, while the study included firms from multiple industries, the sample was dominated by manufacturing and IT organizations, which may limit the applicability of results to less technologically advanced sectors. Fifth, although the sample size of 120 is acceptable for PLS-SEM and the proposed model, for more precision and generalizability of the estimates, sample size should be increased. Finally, the study examined analytics culture as the sole moderating factor; however, other organizational enablers such as leadership commitment, data governance, and technological readiness were not explored and may also significantly shape the effectiveness of big data analytics in achieving superior performance.

## 8 Future research

Future research could address these limitations and further enrich the understanding of big data analytics in B2B contexts. Cross-cultural comparative studies would be valuable in identifying how regional and institutional factors influence analytics adoption and its impact on performance. Longitudinal research designs could examine the temporal progression of analytics maturity and its sustained influence on strategic outcomes. Moreover, future investigations could focus on industry-specific analyses to determine whether the mechanisms linking big data analytics, customer satisfaction, and firm performance differ across sectors such as healthcare, education, or logistics. Firm size also influences these relationships, as previous studies suggest that the development and implementation of big data strategies vary by organizational scale and resource availability (Badghish and Soomro, 2024; Mikalef et al., 2019). Another promising avenue involves exploring the integration of artificial intelligence, machine learning, and predictive analytics to assess how emerging technologies enhance or transform the value derived from big data. Finally, incorporating human and organizational dimensions such as leadership support, data literacy, employee attitudes, and change management, these variables could provide a more comprehensive understanding of the social and cultural foundations that enable data-driven success in B2B organizations.

## Data Availability

The original contributions presented in the study are included in the article/supplementary material, further inquiries can be directed to the corresponding author.
